# Clinical Significance of Programmed Death Ligand‑1 and Intra-Tumoral CD8^+^ T Lymphocytes in Ovarian Carcinosarcoma

**DOI:** 10.1371/journal.pone.0170879

**Published:** 2017-01-26

**Authors:** Jun Zhu, Hao Wen, Xingzhu Ju, Rui Bi, Wenjia Zuo, Xiaohua Wu

**Affiliations:** 1 Department of Gynecologic Oncology, Fudan University Shanghai Cancer Center, Shanghai, PR China; 2 Department of Pathology, Fudan University Shanghai Cancer Center, Shanghai, PR China; 3 Department of Breast Cancer, Fudan University Shanghai Cancer Center, Shanghai, PR China; 4 Department of Oncology, Shanghai Medical College, Fudan University, Shanghai, China; Mayo Clinic College of Medicine, UNITED STATES

## Abstract

Ovarian carcinosarcoma (OCS) accounts for high mortality and lacks effective therapeutic methods. So far, we lack reliable biomarkers capable of predicting the risk of aggressive course of the disease. Programmed death ligand-1 (PD-L1) is expressed in various tumors, and antibodies targeting its receptor programmed cell death 1 (PD-1) are emerging cancer therapeutics. This study was designed to evaluate the expression of PD-L1 and intratumoral CD8+ T lymphocytes by immunohistochemistry from 19 OCS patients who underwent primary surgery at Fudan University Shanghai Cancer Center. The correlations between PD-L1 expression and CD8+ T lymphocytes as well as the patients’ clinicopathologic characteristics were integrated and statistically analyzed. PD-L1-positive expression was observed in 52.6% of intraepithelial tissues and 47.4% of mesenchymal tissues (p = 0.370). Meanwhile, intraepithelial and mesenchymal CD8+ T lymphocytes were positive in 36.8% and 84.2% of OCS, respectively (p = 0.628). A significantly negative correlation was found between mesenchymal CD8+ T lymphocytes and PD-L1 expression (r = -0.630, p = 0.011). Intraepithelial PD-L1-positive expression was associated only with positive ascitic fluid (p = 0.008). Mesenchymal PD-L1-positive patients had a poorer survival than those with negative expression (p = 0.036). Meanwhile, intraepithelial PD-L1-positive patients had a better survival trend than PD-L1-negative patients, though no statistical significance was found (p = 0.061). There was a better postoperative survival noted in mesenchymal CD8-positive patients (p = 0.024), and allthough a better trend of OS was observed in intraepithelial CD8-positive patients, no statistical significance was found (p = 0.382). Positive tumoral CD8+ T lymphocytes and mesenchymal PD-L1-negative expression seem to be associated with better survival in OCS. It is possible that immunotherapy targeting PD-L1 pathway could be used in OCS.

## Introduction

Ovarian carcinosarcoma (OCS), also referred to as ovarian mixed Müllerian tumor, is a rare but aggressive malignancy, occurring in only 1% to 3% of all ovarian cancers [1]. OCS is histologically composed of malignant epithelial and mesenchymal components and classified according to the homologous or heterologous derivation of the mesenchymal tissue in their stromal element. Compared with other ovarian carcinoma, OCS displays an aggressive clinical behavior resulting in poorer survival for both local and distant disease. Moreover, the prognosis of OCS is worse than that of high-grade ovarian carcinomas with a similar stage [2]. Given the rarity and pathological diversity, there is no standard treatment modality for OCS at present. Maximal cytoreduction is still the mainstay therapy for this tumor. In the absence of randomized data, chemotherapy options for patients with OCS are based primarily on data from other ovarian and sarcoma subtypes, as well as retrospective data. Several studies have compared the outcomes between patients treated with platinum-taxane combinations and ifosfamide-based regimens, however, the results remained controversial [3–6]. Nevertheless, the response rates and clinical benefit of adjuvant chemotherapy remains inferior to that of epithelial ovarian carcinoma. Accordingly, there is a pressing effort to optimize the outcome of this generally poor-prognosis cancer by exploring molecular biomarkers that can provide accurate prognosis and targeted therapy.

Tumor-induced immune suppression is a key problem that not only promotes tumor development, but also inhibits the efficiency of anti-tumor treatment. One of the major molecular regulators of tumor immune escape is programmed cell death ligand 1 (PD-L1, CD274, B7-H1), a cell-surface protein induced on T cells, B cells, and monocytes on activation, which may contribute to could help tumor cells immune evasion in combination with its immonomodulatory properties [7]. PD-L1 is expressed on many tumor-infiltrating CD8+ T cells, as well as CD4+ T cells, natural killer (NK) T cells, B cells, dendritic cells, and macrophages[8]. PD-L1 on tumors or antigen-presenting cells in tumor microenvironment has been proposed to promote tumor growth and induce apoptosis of tumor-reactive T cells expressing PD-1 [9]. Blockage of PD-L1 expression on tumor cells might activate tumor-specific T cell to kill tumor cells by mediating tumor necrosis factor alpha (TNF-α) and interferon gamma (IFN-γ) [10, 11]. Furthermore, studies showed that PD-L1 expression is described to have a negative correlation with the density of intratumoral CD8+ T cells[12, 13].

In the present study, we focused our work on the investigation of PD-L1 expression and tumoral CD8+ T lymphocyte count, and their correlations with clinicopathologic features in OCS, in order to further determine their effect on the prognosis of OCS patients.

## Materials and Methods

### Patients and Samples

Paraffin-embedded tissue blocks of 19 OCS patients who had undergone primary surgery were selected from the archival collections (between January 2007 to December 2013) of the Department of Pathology at Fudan University Shanghai Cancer Center, on the basis of adequate tissue for immunohistochemical evaluation. Patients who received neoadjuvant chemotherapy or radiotherapy, underwent incurable surgery, or had multiple cancers, were excluded. The detailed clinical data were collected by retrospective reviews of the patients’ medical charts. In order to confirm the diagnosis, all the microscopic slides were reviewed by the same gynecology-dedicated pathologist (Y.Wu) and confirmed by a second experienced gynecologic pathologist (R.Bi), both were blinded to the original diagnosis. The histologic subtype was classified according to the World Health Organization (WHO) definitions[14].

All the patients in the study cohort provided informed consent. The study was approved by the ethics committee at Fudan University Shanghai Cancer Center, Shanghai, China. All of the participants provided written informed consent for the use of their tissue samples.

Patients were staged using the International Federation of Gynecology and Obstetrics (FIGO) 2012 staging system and were further divided into early stage (I-II) and advanced stage (III-IV) for the purpose of statistical analysis. Operations were performed by gynecologic oncologist to achieve optimal cytoreduction, which was defined as residual disease less than (or including) 1 cm.

### Immunohistochemistry

Immunohistochemistry for PD-L1 was evaluated as previously described [15]. Formalin-fixed, paraffin-embedded tissue sections were immunohistochemically stained. To identify PD-L1, rabbit anti-PD-L1 monoclonal antibody (ab205921, Abcam, Cambridge, MA, 1:50 dilution ratio) was used. The tissue sections were deparaffinized in xylene (2×20 min) and subjected to a graded alcohol dehydration (95%, 90%, 80%, and 70%) to water. Heat-mediated antigen retrieval was performed with EDTA buffer (pH 8) by microwaves for 23 minutes. To block endogenous peroxidase activity, all sections were treated with 100% methanol containing 0.3% H_2_O_2_ at room temperature for 25 minutes. The sections were incubated with rabbit anti-PD-L1 monoclonal Ab overnight at 4°C. Then, the sections were incubated with a biotinylated goat-anti-rabbit secondary Ab (K5007, Dako), followed by an incubation with a streptavidin-peroxidase complex solution at room temperature for 50 min and counterstained with hematoxylin. Positive controls consisted of placenta tissue for the primary antibody. Immunohistochemistry for CD8 (rabbit anti-CD8 mAb, ab93278, Abcam, UK, 1:250 dilution ratio) was conducted according to standard automated methods[16].

### Evaluation of Immunostaining

Two gynecological pathologists (Y.W. and R.B.) who had no knowledge of the patients’ clinical status or outcome independently examined the prepared immunohistochemical slides without any prior information on the clinical history of the patients. Meanwhile, the reviewing pathologists were unaware of PD-L1 findings at the time that the CD8+ T lymphocytes scores were generated. The expression of PD-L1 was evaluated both in cancermatous cell nests (intraepithelial) and in sarcomatous cells nests (mesenchymal). The proportion of PD-L1-positive cells was estimated as a percentage of the total tumor cells. The tumor cells typically showed membranous staining with a variably strong component of cytoplasmic staining. Then, we defined samples with less than 10% of positive cells, 10–50% of positive cells, more than 51% of positive cells as weak, moderate, strong expression respectively. Evaluation was assessed in both the intraepithelial and mesenchymal compartments. For analysis of the expression of PD-L1, samples with a ≥ 10% PD-L1-positive tumor cells were classified as PD-L1-positive [13].

In addition, immunohistochemistry for intratumoral CD8+ T lymphocytes was also evaluated [12]. In brief, intratumoral CD8+ T lymphocytes was separately counted according to localization in the carcinomatous and sarcomatous components. CD8+ T lymphocytes infiltrating into intraepithelial and mesenchymal cell nests were designated carcinomatous and sarcomatous CD8+ T lymphocytes, respectively, and were observed under a microscopic field at ×200 magnification. Three areas with the most abundant infiltration were selected, and the average count was calculated. A cutoff point was used as to divide all tumors into groups as having either negative (fewer than five cells per field) or positive (more than or equal to five cells per field) infiltration in carcinomatous tissue. CD8+ T lymphocytes detected within the mesenchymal component were evaluated in the same manner.

### Statistical Analysis

Statistical comparisons between clinicopathologic features, PD-L1 expression and intratumoral CD8+ T lymphocyte count were evaluated using a Fisher’s exact test. In survival analysis, the starting point was defined as the day on which surgery was performed. The endpoint of OS period was defined as the day on which the patient was confirmed alive or dead, respectively. Estimation of OS was calculated by the Kaplan-Meier method, and the curves were compared using a log-rank test. All of the *P*-values reported were two-sided, and a value of P <0.05 was considered statistically significant. Statistical Package for Social Science (SPSS) statistical software (Version 20.0, SPSS, Inc., Chicago, IL, USA) and GraphPad Prism (Version 5.0, GraphPad Software, Inc., La Jolla, CA, USA) were used for all of the analyses.

## Results

### Demographics and Clinicopathological Characteristics

Demographics and clinicopathological characteristics are summarized in Table 1. The average age of the patients was 58.5 years (range, 32–75; SD, 11.1 years). Of the 19 OCS patients, 2 (10.5%) were diagnosed as stage I, 5 (26.3%) were diagnosed as stage II, and 12 (63.2%) were diagnosed as stage III. Histological subtypes of the tumor comprised 8 (42.1%) cases of homologous and 10 (52.6%) cases of heterologous subtypes.

**Table 1:** Demographic and clinicopathological characteristics of 19 OCS patients.

Demographic and Pathologic Features	N	Death (%)	P-Value
Age at diagnosis			
Median (range)	58 (32–75)		NA
Mean (SD)	58.5 (11.1)		
Family history of malignancy			0.607
Y	14	7 (50.0)	
N	5	4 (80.0)	
FIGO stage			0.153
I	2	0 (0.0)	
II	5	2 (40.0)	
III	12	9 (75.0)	
Menopause			
Y	15	9 (60.0)	0.996
N	4	2 (50.0)	
BMI			0.438
< 25	10	5 (50.0)	
≥ 25	9	6 (66.7)	
Pretreatment CA-125			0.948
≤ 200 U/ml	4	2 (50.0)	
> 200 U/ml	14	9 (64.3)	
Ascitic fluid			0.089
Negative	9	6 (66.7)	
Positive	10	5 (50.0)	
Bilateral tumors			0.027
N	14	6 (42.9)	
Y	5	5 (100.0)	
Tumor size, cm			0.286
≤ 12	11	7 (63.6)	
> 12	8	4 (50.0)	
Mesenchymal component			0.111
Homologous	9	5 (55.6)	
Heterologous	10	6 (60.0)	
Residual disease			0.019
NVD	6	2 (33.3)	
≤ 1cm	6	2 (33.3)	
> 1cm	7	7 (100.0)	
Adjuvant chemotherapy			0.367
Y	16	8 (50.0)	
N	3	3 (100.0)	

NVD = no visible disease.

**Fig 1:**
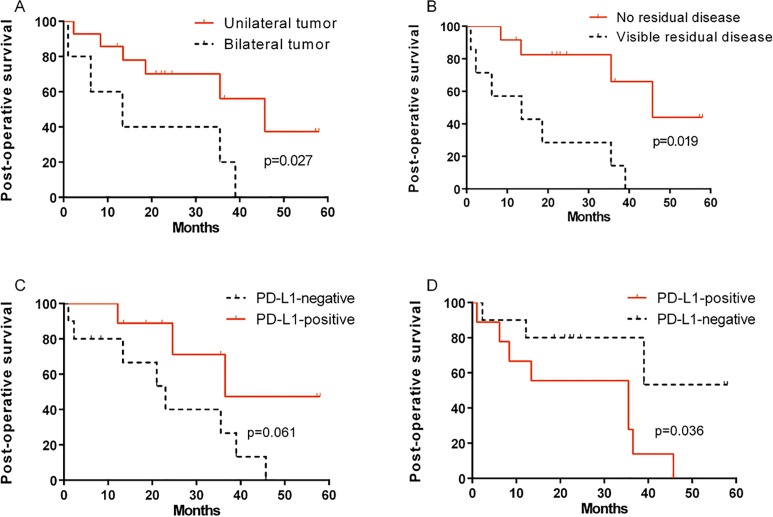
Kaplan-Meier survival curves of patients with ovarian carcinosarcoma (OCS). Fig 1A and 1B show the post-operative survival classified according to tumor site (unilateral tumor vs. bilateral tumors) and residual disease (no visible disease vs. visible residual disease), respectively. Patients with bilateral tumors and visible residual disease showed significantly worse prognosis (p = 0.019 and p = 0.027, log-rank test). Fig 1C and 1D show the post operation survival curves between mesenchymal PD-L1-positive and PD-L1-negative expression. Intraepithelial PD-L1-positive patients had a trend of better survival than PD-L1-negative patients (p = 0.061). A significant difference in postoperative prognosis between mesenchymal PD-L1-positive and PD-L1-negative patients was observed (p = 0.036).

At the end of study, 11 (57.9%) patients had died due to the disease, and 8 (42.1%) survived. The median follow-up period was 22.2 months (range, 1.0–58.0 months). The median overall survival was 35.5 months (range, 10.6–60.4 months) and the overall 3-year survival rate was only 44.3%. By univariate analysis, there was a statistically significant difference between the survival distributions of unilateral tumor group and bilateral tumor group (Fig 1A, p = 0.027, log-rank test). In addition, a statistically significant difference in survival distribution was observed between patients with no visible disease and those with visible residual disease (Fig 1B, p = 0.019, log-rank test). The results of statistical survival analysis for clinicopathological variables are summarized in Table 1.

### PD-L1 Expression and Intratumoral CD8+ T Lymphocytes in OCS

**Fig 2:**
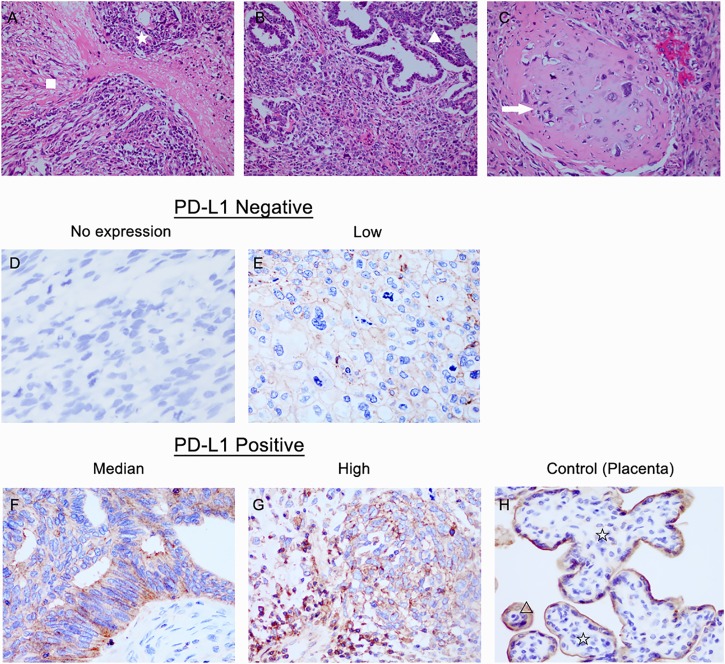
Immunohistochemical staining for programmed death ligand 1 (PD-L1) in intraepithelial and mesenchymal tumor tissue. A-C: Haematoxylin and eosin-prepared samples of OCS tissues containing both carcinomatous (☆) and mesenchymal components (ϒ). The homologous subtype is composed of high-grade undifferentiated round cell or spindle cell sarcomatous proliferation (tissue native to the ovary, △). Heterologous carcinosarcoma may show cartilaginous differentiation (tissue not native to the ovary, arrow). D-F: Representative immunohistochemical staining for PD-L1 expression in OCS. Samples were divided into three groups according to the intensity of PD-L1 expression: weak (D), moderate (E), and strong (F) staining of PD-L1. Tissue of placenta (G) was used as positive control for PD-L1 expression (at x400 magnification).

**Fig 3:**
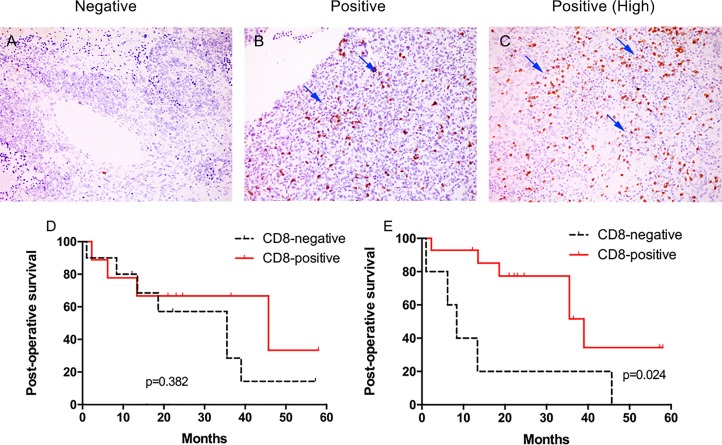
**Representative immunohistochemical staining for CD8+ T lymphocytes in tumor tissue: negative (A) and positive (B&C) (arrows indicate intratumoral CD8+ T cells,at x200 magnification)**. Kaplan-Meier analysis of tumoral CD8+ T lymphocytes and survival: (D) Post operation survival curves between CD8-positive and CD8-negative intraepithelial tissues. (E) Post operation survival curves between CD8-positive and CD8-negative mesenchymal tissues.

Immunohistochemical PD-L1 expression and intratumoral CD8+ T lymphocytes of OCS tissues are depicted in Figs 2 and 3. Among the 19 tissue samples, 10 (52.6%) were positive for PD-L1 expression and 9 (47.4%) were negative in the intraepithelial components. In the mesenchymal components, PD-L1-positive and PD-L1-negative were observed in 9 (47.4%) and 10 (52.6%) cases, respectively. There were no significant difference in the expression of PD-L1 between the intraepithelial and mesenchymal components (P = 0.370).

CD8-positive was observed in 9 (47.4%) cases in the intraepithelial component, and 10 (52.6%) cases were CD8-negative. In the mesenchymal component, CD8-positive and CD8-negative were observed in 14 (73.7%) and 5 (26.3%) tissues respectively (Fig 2). No significant difference was found between the expression of intraepithelial and mesenchymal CD8+ T lymphocytes count (p = 0.628).

### Association of PD-L1 Expression and Tumoral CD8+ T Lymphocytes with Clinicopathologic Characteristics

As shown in Table 2, intraepithelial PD-L1-positivity was significantly associated with positive ascite fluid (25% vs.85.7%, p = 0.020). There were no associations between intraepithelial or mesenchymal PD-L1 expression and any remaining clinicopathologic characteristics. Also, no correlations were found between intraepithelial or mesenchymal CD8+ T lymphocytes and various clinicopathologic factors.

**Table 2:** Correlations between the expression of PD-Ls or tumor-infiltrating CD8+ lyphocytes and clinicopathological characteristics in ovarian carcinosarcoma (*n* = 19).

Variable	Intraepithelial PD-L1 expression				Mesenchymal PD-L1 expression			Intraepithelial CD8+ TIL			Mesenchymal CD8 + TIL		
	*n*	Negative	Positive	*P*	Negative	Positive	*P*	Negative	Positive	*P*	Negative	Positive	*P*
Age				0.605			0.650			1.000			0.338
<58	8	4	4		5	3		4	4		1	7	
≥58	11	6	5		5	6		6	5		4	7	
Stage				0.800			0.528			0.528			1.000
I	2	1	1		2	0		0	2		0	2	
II	5	2	3		3	2		3	2		1	4	
III	12	7	5		5	7		7	5		4	8	
Histology subtype				0.996			0.998			0.656			1.000
Homologous	9	5	4		5	4		4	5		2	7	
Heterologous	10	5	5		5	5		6	4		3	7	
Menopause status				0.087			0.906			0.303			1.000
Premenopausal	4	4	0		2	2		1	3		1	3	
Postmenopausal	15	6	9		8	7		9	6		4	11	
Pretreatment CA125				0.588			1.000			1.000			1.000
≤ 200 U/ml	4	3	1		2	2		2	2		1	3	
> 200 U/ml	14	7	7		7	7		8	6		4	10	
Ascitic fluid				0.020			0.650			1.000			0.603
Negative	12	9	3		7	5		6	6		4	8	
Positive	7	1	6		3	4		4	3		1	6	
Tumor site				0.303			0.141			1.000			0.084
Unilateral	14	6	8		9	5		7	7		2	12	
Bilateral	5	4	1		1	4		3	2		3	2	
Tumor volume				0.370			0.650			1.000			0.338
≤ 12 cm	11	7	4		5	6		6	5		4	7	
> 12cm	8	3	5		5	3		4	4		1	7	
Residual tumor				0.350			0.650			0.350			1.000
R0	12	5	7		7	5		5	7		3	9	
Visible residual disease	7	5	2		3	4		5	2		2	5	
Chemotherapy				0.608			0.608			1.000			1.000
PTX+CBP	10	4	6		4	6		5	5		3	7	
IFO/DOX	5	3	2		3	2		3	2		2	3	

R0 = no visible disease after cytoreductive surgery. PTX = Paclitaxel; CBP = Carboplatin; IFO = Ifosfamide; DOX = Doxorubicin

### Correlation Between PD-L1 Expression and Outcome

Of the 19 patients, intraepithelial PD-L1-positive patients had a trend of better survival than PD-L1-negative patients (p = 0.061, Fig 1C). Three-year postoperative survival rate of intraepithelial PD-L1-positive and PD-L1-negative patients was 56.3% and 33.3%, respectively. On the other hand, a significant difference in postoperative prognosis between mesenchymal PD-L1-positive and PD-L1-negative patients was observed (p = 0.036; Fig 1D). Three-year postoperative survival rate of mesenchymal PD-L1-positive and PD-L1-negative patients were 22.2% and 78.8%, respectively.

Subgroup analysis noted significant differences in 3-year survival rate between intraepithelial PD-L1-positive and PD-L1-negative patients when categorized by positive ascite fluid (62.5% vs. 0%, p = 0.014). In addition, there were significant differences in 3-year survival rate between mesenchymal PD-L1-positive and PD-L1-negative patients when categorized by the following variables (Table 3): tumor stage (I-II; 0% vs. 100%, p = 0.034), heterologous subtype (0% vs. 75.0%, p = 0.048), small tumor volume (0% vs. 50.0%, p = 0.004).

**Table 3:** Three-year survival rate of 19 OCS patients according to clinicopathologic characteristics and tumor PD-L1 status (Log-rank).

Variable	n	Intraepithelial			Mesenchymal		
		PD-L1 positive, Death (%)	PD-L1 negative, Death (%)	*P*	PD-L1 positive, Death (%)	PD-L1 negative, Death (%)	*P*
Tumor stage							
Early stages (I-II)	7	1/4 (25.0)	1/3 (33.3)	0.248	2/2 (100)	0/5 (0)	0.034
Advanced stages (III-IV)	12	2/5 (40.0)	7/7 (100)	0.357	6/7 (85.7)	3/5 (60.0)	0.814
Ascite fluid							
Positive	7	2/6 (33.3)	1/1 (100)	0.014	3/4 (75.0)	0/3 (0)	0.098
Negative	12	1/3 (33.3)	7/9 (77.8)	0.662	5/5 (100)	3/7 (42.9)	0.416
Histologic subtype							
Homologous	9	1/4 (25.0)	4/5 (80.0)	0.103	3/4 (75.0)	2/5 (40.0)	0.366
Heterologous	10	2/5 (40.0)	4/5 (80.0)	0.415	5/5 (100)	1/5 (25.0)	0.048
Tumor site							
Unilateral	14	2/8 (25.0)	4/6 (66.7)	0.155	4/5 (80.0)	2/9 (22.2)	0.156
Bilateral	5	1/1 (100)	4/4 (100)	0.754	4/4 (100)	1/1 (100)	0.156
Tumor volume (diameter)							
≤ 12cm	11	2/4 (50.0)	5/7 (71.4)	0.315	6/6 (100)	1/5 (20.0)	0.004
> 12cm	8	1/5 (20.0)	3/3 (100)	0.167	2/3 (66.7)	2/5 (40.0)	0.955

### Correlation Between CD8+ T Lymphocytes and Outcome

Among the 19 patients, both intraepithelial and mesenchymal CD8-positive patients showed a better postoperative survival than intraepithelial and mesenchymal CD8-negative patients (p = 0.382, Fig 3D and p = 0.024, Fig 3E, respectively). Three-year postoperative survival rate of intraepithelial CD8-positive and CD8-negative patients was 66.7% and 28.6%, respectively. Meanwhile, three-year postoperative survival rate of mesenchymal CD8-positive and CD8-negative patients was 51.6% and 20.0%, respectively. While the mesenchymal three-year postoperative survival rate of CD8-positive patients with unilateral tumor and bilatral tumors was 55.0% and 50.0%, respectively (p = 0.524). The three-year postoperative survival rate of CD8-negative patients with unilateral tumor and bilatral tumors was 50.0% and 0%, respectively (p = 0.277). Though of no significance, we did observe a trend towards better survival in unilateral tumor compared to bilateral tumors in both mesenchymal CD8-positive and negative cases. These data provide evidence of the contribution of the immune system in the eradication of ovarian carcinosarcomas.

There were no significant differences noted in 3-year survival rate between intraepithelial CD8-positive and CD8-negative cases and other clinicopathologic characteristics including tumor stage, ascitic status, histologic subtype, tumor site and tumor volume (Table 4). On the other hand, subgroup analysis indicated that significant differences in 3-year survival rate between mesenchymal CD8-positive and CD8-negative patients when categorized by the following variables: early tumor stage (66.7% vs. 0%, p = 0.014), positive ascite fluid (62.5% vs. 0%, p = 0.014), heterologous subtype (0% vs. 75.0%, p = 0.048), small tumor volume (55.6% vs. 0%, p = 0.001) and bilateral tumors (50.0% vs. 0%, p = 0.063).

**Table 4:** Three-year survival rate of 19 OCS patients according to clinicopathologic characteristics and tumor CD8+ T lymphocytes (Log-rank).

Variable	n	Intraepithelial			Mesenchymal		
		CD8 positive, Death (%)	CD8 negative, Death (%)	*P*	CD8 positive, Death (%)	CD8 negative, Death (%)	*P*
Tumor stage							
Early stages (I-II)	7	0/4 (0)	2/3 (66.7)	0.186	1/6 (16.7)	1/1 (100)	0.014
Advanced stages (III-IV)	12	4/5 (80.0)	5/7 (71.4)	0.847	5/8 (62.5)	4/4 (100)	0.524
Ascite fluid							
Positive	7	1/3 (33.3)	2/4 (50.0)	0.757	2/6 (33.3)	1/1 (100)	0.014
Negative	12	3/6 (50.0)	5/6 (83.3)	0.286	4/8 (50.0)	4/4 (100)	0.449
Histologic subtype							
Homologous	9	2/5 (40.0)	3/4 (75.0)	0.513	3/7 (42.9)	2/2 (100)	0.415
Heterologous	10	2/4 (50.0)	4/6 (66.7)	0.995	3/7 (42.9)	3/3 (100)	ND
Tumor site							
Unilateral	14	4/7 (57.1)	2/7 (28.6)	0.254	4/12 (33.3)	2/2 (100)	0.276
Bilateral	5	2/2 (100)	3/3 (100)	0.364	2/2 (100)	3/3 (100)	0.063
Tumor volume (diameter)							
≤ 12cm	11	2/5 (40.0)	5/6 (83.3)	0.319	3/7 (42.9)	4/4 (100)	0.001
> 12cm	8	2/4 (50.0)	2/4 (50.0)	0.943	3/7 (42.9)	1/1 (100)	0.983

ND = not determined.

### Correlation Between PD-L1 Expression and CD8+ T Lymphocytes

We then analyzed the correlation between tumor PD-L1 expression and intratumoral CD8+ T lymphocyte count. In mesenchymal components, there was a significantly negative correlation between PD-L1 expression and CD8+ T lymphocyte (r = -0.630, p = 0.011; Table 5). However, there was no significant correlation found in intraepithelial PD-L1 expression and CD8+ T lymphocyte count (r = -0.267, p = 0.370). It is likely that PD-L1 on tumor cells inhibits the mesenchymal invasions of tumor-specific CD8+ T cells, resulting in tumor evasion from the immune system.

**Table 5:** Correlation between tumor PD-L1 expression and intratumoral CD8+ T lymphocyte count.

**Fig**	**CD8 (Intraepithelial)**	
	**Negative**	**Positive**
Negative	4	6
Positive	6	3
**PD-L1 (Mesenchymal)**	**CD8 (Mesenchymal)**	
	**Negative**	**Positive**
Negative	0	10
Positive	5	4

## Discussion

Carcinosarcoma of gynecologic origin is a rare but aggressive tumor, characterized by an admixture of epithelial and mesenchymal elements. Although surgical debulking followed by adjuvant chemotherapy remains the mainstay of treatment for ovarian carcinosarcoma, low response rates and high recurrence rate is a major concern for the majority of patients [2]. Moreover, the five-year survival rate for OCS is 26.63% compared to 43.61% for serous carcinomas [17]. Cancer immunotherapy is now rapidly evolving with clinical benefits targeting the PD-1/PD-L1 pathways. Blocking of PD-L1 with monoclonal antibodies could trigger anti-tumor immune responses. It has been suggested that tumoral PD-L1 expression may be an adequate biomarker to predict immunotherapeutic responsiveness in many solid tumors. In this study we successfully revealed a negative correlation between mesenchymal CD8+ T lymphocytes and PD-L1 expression. Meanwhile, the prognostic value of CD8+ T lymphocytes and PD-L1 expression were particularly remarkable in mesenchymal component in OCS.

The expression of PD-L1 has been investigated in various malignancies. However, very limited data have been reported in ovarian carcinoma, with no specific study reported in OCS alone. In our present study, 52.6% of intraepithelial component and 47.4% of mesenchymal component was observed to be PD-L1-positive. Hamanishi et al [12] reported the proportion of PD-L1 high expression to be 68.6% in 70 cases of ovarian cancers, including 28 serous carcinomas, 22 clear cell carcinomas, 11 endometrioid carcinomas, 2 mucinous carcinomas, and 7 other types of ovarian cancer. Darb-Esfahani et al [18], however, found a higher proportion of PD-L1 expression (up to 75.7%) in 202 cases of evaluable high grade serous ovarian cancer. The discrepancy between the aforementioned studies and our study could be due to different antibodies used (the antibodies used in previous studies may have had lower specificity), and the difference in scoring strategies used to evaluate PD-L1 expression. Like most previous studies on PD-L1 expression, we chose a 5% membranous staining as a cutoff for PD-L1-positive expression [19, 20].

There have been conflicting results in the literature as to whether PD-L1 expression is a favorable or adverse prognostic factor. Previous studies have shown that PD-L1 expression was correlated with unfavorable prognosis in non-small lung cancers, colorectal and breast cancers[21–23]. However, other studies revealed a better OS in malignancies with high expression of PD-L1 [24, 25]. Our results, showing a positive trend between intraepithelial PD-L1 expression and outcome, align with a recent study (Esfahani et al.[18]) which assessed the expression of PD-L1 in high grade serous ovarian cancer (HGSOC).They also confirmed the significant positive prognostic impact of PD-1 and PD-L1 mRNA expression. On the contrary, our study revealed the negative effect of mesenchymal PD-L1 expression on survival, which was in line with the findings of Shen et al.[26] who also demonstrated a slight trend for poorer overall survival in osteosarcoma patients with high PD-L1 expression. Though Hamanishi et al.[12] demonstrated that tumor PD-L1 expression was an independent predictor of poor prognosis in ovarian cancer, their study included different histological subtypes of ovarian cancers and only 40% of the cohort consisted of serous carcinomas, therefore those results are hard to compare with other studies. The discrepant results of intraepithelial and mesenchymal PD-L1 expression on survival in our study could be explained by the fact that the epithelial tumor cells dictate metastatic behaviors [27] and the sarcomatous component predominates the prognosis. Similarly, a separate study by Abiko et al.[28] demonstrated that high expression of PD-L1 is associated with increased invasiveness and peritoneal dissemination in epithelial ovarian cancer. In addition, F.Amant et al.[29] demonstrated a preponderance of epithelial cells in the primary setting, and that the sarcomatous cells dominated the tumorigenic cell population in the recurrent setting at interval debulking. These results suggested that the epithelial component drives the tumor, while the sarcomatous component predominates the recurrent setting, by extension, the survival of patients.

PD-L1 pathway plays an important role in the suppression of T cell activation, allowing cancer cells to evade host immune surveillance [30, 31]. Hamanishi et al.[12] demonstrated that PD-L1 expression was inversely correlated with CD8+ T lymphocytes in ovarian cancers. Studies in animals models also demonstrate that PD-L1 on tumor cells inhibits T-cell activation, decreasing the ability of T cell to kill tumor cells[12, 32]. Although it was not significant, the density of TILs in PD-L1-positive tumor regions were lower than that in PD-L1-negative regions. We found a negative correlation between mesenchymal CD8+ T lymphocytes and PD-L1 expression, and uncovered that this small subpopulation of T cells has a biological significance which can be measured by patient prognosis. These results suggest that the expression of PD-L1 on tumor cells may contribute to negative regulatory immune responses against TILs in OCS.

The findings that mesenchymal PD-L1 and CD8+ cells are significantly associated with survival in early tumor stages or small tumor volume subgroups are intriguing. This might be explained by the fact that the differentiation of naive tumor-specific T cells begins early during tumor development. In early stage disease, CD8+ T cells recognize tumor-specific antigens, and trigger the anti-tumor immune responses. If tumor antigen persists in advanced diseases, tumor-infiltrating cells become increasingly dysfunctional with high levels of PD-L1 expression, and failing to produce effector cytokines in response to antigens. Bernhard and colleagues revealed a negative correlation between the density of intratumoral CD8 and the tumor stage. CD8 cell densities decreased gradually with the tumor extension into the primary organ[33]. Moreover, CD8+ cells in specific tumor regions was a useful criterion for the prediction of tumor recurrence and survival in early-stage patients[34–36]. It is possible that tumor infiltration by cytotoxic T lymphocytes could reflect the level of antitumor immune response. A specific localized immune reaction could, in part, influence the progression of the tumor. In addition, although no significance was found, a trend towards better survival was observed in patients with unilateral tumor compared to bilateral tumors. The presence of bilateral tumors suggests the possibility of metastatic tumor from another side and the extension of tumor bilology[37]. The decreased immune cell densities in advanced disease could indicate a progressive immune escape, and the magnitude of the immune reaction at the early stage of tumor could be a major determinant for controlling the tumor evolution. Accordingly, features observed in minimal tumor burden as well as in early tumor stages may provide a novel indicator to better predict of tumor characteristics.

Anti-PD-L1 therapy has demonstrated durable responses in a number of different advanced malignancies. A phase I trial (NCT00729664) of BMS-936559, an an-ti-PD-L1 agent, showed that the overall responses (ORs) were observed in patients with melanoma (MEL, 9/52), renal cell carci-noma (RCC, 2/17), NSCLC (5/49), and ovarian cancer (1/17). In 16 patients with ≥1 year of follow-up, stable disease (SD, ≥ 24 weeks) was observed in 14 patients with MEL, six patients with NSCLC, three patients with ovarian cancer, and seven patients with RCC[38]. Nivolumab, an anti-PD-1 antibody, demonstrated an efficacy in a proportion of relapsed platinum-resistant ovarian cancer patients, with a median progression-free survival time of 3.5 months (95% CI, 1.7 to 3.9 months), and a median overall survival time of 20.0 months[39]. An underlying explanation for the relatively low overall control rate may be that patients whose tumors lack preexisting TILs do not respond to PD-1/PD-L1 blockade or combinations that primarily focus on boosting T-cell effector functions. Studies in mice model also showed that response to PD-L1 blockade required a preexisting adaptive immunity in tumors, and tumors without TILs failed to respond [40]. It is possible that in cancer tissues without lymphocyte infiltration, PD-1/PD-L1 blockade is unlikely to be sufficient, and combinations will be required to mount an anti-tumor adaptive immune response.

There are several limitations to this study. The cohort only includes a small number of patients, therefor the results lack the ability to perform multivariate analysis. A further retrospective study utilizing a larger patient cohort may provide a better understanding of the impact of PD-L1 and TILs in OCS. Given the retrospective nature of the analysis and significant results being confined to subgroups, caution must be exercised over-interpreting the results.

In conclusion, our current findings demonstrated that PD-L1 expression, and CD8+ T lymphocytes in particular, are biologically and clinically important in OCS. To our knowledge, this is the first report measuring PD-L1 expression and its correlation with CD8+ T lymphocytes and clinicopathologic and prognostic characteristics in OCS. Importantly, the data presented here could also provide a rationale to help identify which patients are more likely to relapse and who might benefit from additional therapy. Our findings are a thrilling first step in determining the role of PD-L1 in OCS, further studies on cancer cell lines models would be done to confirm its specific functions, which could help improve and add value to the current findings. We assume that the PD-L1 inhibitory pathway, in addition to regulating T-cell responses to self-antigens and viral antigens, may also regulate tumor-infiltrating CD8+ T-cell responses in OCS. In view of our results, PD-L1 may therefore be used as a new target in designing T cell-based immunotherapy for human cancers.

## Supporting Information

S1 Table Clinicopathologic characteristics and survival outcome of 19 OCS patients. (DOCX)
