# Supplementary material for: Clinical Significance of Programmed Death Ligand‑1 and Intra-Tumoral CD8+ T Lymphocytes in Ovarian Carcinosarcoma
Source: PLoS One. 2017 Jan 26;12(1):e0170879. doi: 10.1371/journal.pone.0170879 (PMC5268655; doi:10.1371/journal.pone.0170879)
Supplement: S1 Table — (DOCX) [file pone.0170879.s001.docx]

| Supplementary Table 1. Clinicopathologic characteristics and survival outcome of 19 OCS patients. | | | | | | | | |
| --- | --- | --- | --- | --- | --- | --- | --- | --- |
| Case | Age (years) | Stage (FIGO) | Sarcomatous  component | Intraepithelial PD-L1 | Mesenchymal PD-L1 | Intraepithelial CD8+ T lymphocyte | Mesenchymal CD8+ T lymphocyte | Follow-up  (months) |
| 1 | 65 | III | Homologous | Negative | Negative | Positive | Positive | D, 2.3 |
| 2 | 50 | II | Heterologous (R) | Negative | Negative | Positive | Positive | A, 21 |
| 3 | 48 | I | Homologous | Negative | Negative | Positive | Positive | A, 23 |
| 4 | 57 | II | Homologous | Positive | Negative | Negative | Positive | A, 57.3 |
| 5 | 57 | III | Heterologous (R) | Positive | Negative | Negative | Positive | A, 22.2 |
| 6 | 57 | III | Heterologous (C) | Positive | Negative | Negative | Positive | A, 12.2 |
| 7 | 74 | III | Heterologous (C,R) | Positive | Negative | Negative | Positive | D, 18.6 |
| 8 | 65 | II | Heterologous (R) | Positive | Negative | Positive | Positive | A, 24.6 |
| 9 | 58 | I | Homologous | Positive | Negative | Positive | Positive | A, 58 |
| 10 | 68 | II | Homologous | Negative | Positive | Negative | Negative | D, 8.4 |
| 11 | 75 | III | Homologous | Negative | Positive | Positive | Negative | D, 45.7 |
| 12 | 32 | III | Heterologous (R) | Negative | Positive | Negative | Positive | D, 35.5 |
| 13 | 58 | III | Heterologous (R) | Positive | Positive | Negative | Positive | D, 13.5 |
| 14 | 50 | III | Homologous | Positive | Positive | Positive | Positive | A, 36.5 |
| 15 | 74 | III | Homologous | Negative | Negative | Negative | Positive | D, 39.0 |
| 16 | 60 | III | Heterologous (R) | Negative | Positive | Negative | Negative | D, 1.0 |
| 17 | 41 | III | Heterologous (C) | Negative | Positive | Positive | Negative | D, 13.4 |
| 18 | 58 | III | Heterologous (R) | Negative | Positive | Positive | Negative | D, 6.2 |
| 19 | 64 | II | Homologous | Positive | Positive | Negative | Positive | D, 35.5 |
| R, rhabdomyosarcoma; C, chondrosarcoma; PD-L1, Programmed death ligand 1; A, alive; D, dead of disease. | | | | | | | | |
